# Supplementary material for: The regulatory effect of herd structure on pig production under the environmental regulation
Source: PLoS One. 2022 Apr 26;17(4):e0266687. doi: 10.1371/journal.pone.0266687 (PMC9042276; doi:10.1371/journal.pone.0266687)
Supplement: Attachment — Submitted filename: 20220118Amendments to each review comment.docx [file pone.0266687.s001.docx]

Amendments to each review comment:

Thank you very much for the experts' valuable opinions. We responded to each opinion one by one. The specific opinions and amendments are shown below.

Reviewer #1: To my opinion, your topic is principally interesting and important for the scientific community and eyond.

As I am not a native speaker, I cannot judge the linguistic English quality of the manuscript in every detail. However, I think there is some room for improvement, e.g. in line 142 ff. Another example: in line 170/171, I would write “…believe that they have…” instead of a repetition of “environmental regulations and village regulations and folk conventions”.

Since I am primarily concerned with other methods (life cycle assessment and sustainability analyses), I cannot give a well-informed opinion on the methodological novelty of the study. The calculations and the statistical seem correct.

After the adjustment, this part of the content was deleted, and the content of the article introduction was readjusted. In the adjustment, the expression similar to "... believe they have …" was also reduced.

Your manuscript does not follow the classical structure proposed by scientific journals such as (alos) PLOS One (with Introduction, Material & Methods, Results, Discussion,…). I do not know if this is obligat

The structure of the full text has been adjusted according to the article of RD Internalization, Domestic Technology Alliance, and Innovation in Emerging Market published in June 25, 2021 by PLOS ONE magazine. The current article includes five parts: introduction, theoretical analysis, models and materials, empirical analysis, conclusions and discussion.

Regarding the abstract:

Line 8 (and other places!): Why do you address pollution control only regarding breeding? I know that your modeling focusses on breeding, but at least in European and North American pig production, the fattening phase is the most relevant point, accounting for about three-fourths of the impact over the life cycle (in terms of GHG emissions, among other impacts). Thus, I would rather write “pig production” (or “pig husbandry”?) instead of “breeding” in the Introduction schapter.

The words used in the full text are redefined, including the words of breeding and production, producer and breeding subject, and the full text is processed consistently to reduce the ambiguity in reading.

Line 19: It is difficult to interpret the two early warning values without the description of any unit.

The early warning value is the herd structure, which is the ratio of sows to fattening pigs and has no unit. The meaning of the early warning value is reflected in the text with full reference to expert opinions, but the unit of the value is additionally indicated in the text.

Regarding Introduction:

The chapter well describes the Chinese situation (the application case), however, in comparison to other papers from my discipline, I would have expected some more references to peer reviewed literature and a description of, for instance, another country/continent or the global situation concerning price fluctuations, pests such as the African Swine Fever, etc. To my knowledge, the Chinese pork market is related to the markets of many other countries with specific mass and price fluctuations. Other countries have their own pollution control regulations – how are they interconnected and comparable with Chinese regulations? These aspects are not reflected in your manuscript! But they could be at least considered in a part of your Introduction section and taken up in the Discussion section.

According to the expert's opinion, on the one hand, the introduction and the discussion were revised to include the relevant contents of the environmental regulation policies of animal husbandry in the United States and Europe, for example, the contents of reference 5 and reference 10 in the introduction, and at the same time, the scope and limitations of the research were emphasized in the discussion. As the scope of the research is China's policy and has certain differences with other countries' policy systems, specific suggestions were put forward only for China's policy formulation in the analysis of applicability, which has reference value for other countries.

Regarding Material and Method section:

The first part(s) of your rather long Material and Method section (“Literature review”) seems to me as a part of the Introduction. You wrote a research article, not a review paper.

In my opinion the Material and Methods section in a research article needs to describe all important aspects, which form the basis for your own modelling. To my opinion, the (narrower) Material and Method section should describe the used databases and key words regarding “Literature research” as well as data sources, models (formulas,...) and the variables that are used in the analysis. Your Material and Method section (“Literature review”) describes over long passages more the background of your work.

According to your suggestion, we readjusted the structure of the article, compressed the related contents of introduction, references and theoretical analysis, reduced the number of references, and combed the context of the existing research and the foundation of the model construction of this article with only necessary references.

The chapter (sub-/heading) structure of the manuscript, especially regarding Material & Methods, and the size of some chapters seems somewhat disproportionate.

According to your suggestion, the materials and methods of the article are adjusted and divided into two parts: research method and variable selection. The research method includes policy effectiveness evaluation method and empirical method. In the variable selection, the variables necessary for empirical analysis are sorted out one by one and described statistically.

Line 106: You use the abbreviation “CPI” for one time only. Please write it in full words.

Line 123: The same with the abbreviation “DID”. Please write it in full words.

Furthermore, some abbreviations regarding statistical analysis (i.a. LCC, HP, IPS test) are not given with a full name by the first use.

According to the expert's suggestion, the full names of all the abbreviations used in the text are marked, in which CPI is on line 47 and DID is on line 57.

LLC etc. in line 360.

I like the message from lines 176 to 189 and I think that this would also fit well at the end of the Introduction chapter. Then, before the actual chapter Material & Methods, formulate the hypotheses or research questions!

This part has been integrated into the new introduction and a research hypothesis has been put forward based on theoretical analysis.

I miss a description of the early warning (score) in Material & Methods. This is a value that you have specifically calculated within your paper, isn’t it? Thus I would like a description of how you have used it.

Part of the introduction has been deleted, but the part contributed by this article has been added to the conclusion and discussion. The second part emphasizes the use of herd structure and early warning interval, which can not only early warn the fluctuation of production and realize stable supply, but also realize the expansion of industry by increasing the herd structure.

The description of the Material & Methods chapter (“Theoretical Analysis”; from line 190 onwards) could probably be phrased more concisely (and more "scientifically"), especially from line 205 onwards. For instance, I would delete the sentence with “Although there is a saying that…” or radically shorten it!

Following the expert's advice, we deleted this section to simplify the presentation. At the same time, we simplified other parts, such as 110 lines and 126 to 129 lines.

Line 257-258: Please rephrase the title, e.g. “Pig production factors and environmental regulation” (Is that too much shortened? In any case, I wouldn't use a question-like sentence as a heading).

Generally, I miss a numbering of the headings in your manuscript in order to be able to clearly distinguish their levels.

We have added the serial number of the section in the recent modification, and have compressed the title of the section so as to express the content of this section clearly and concisely.

Line 273, 306 and others: you did not define hypotheses (nor detailed research questions) in the Introduction section, but describe the hypotheses' achievement in the Material & Methods-section (what is not possible!).

We readjusted the structure of the article, put forward the core research issues based on the introduction, and constructed a theoretical model for analysis. Then we put forward the corresponding research assumptions based on the theoretical analysis, and then tested the research assumptions through empirical tests. Such a revision is more in line with the basic paradigm of economic analysis in the article.

For me, the first sentence in the Conclusions is one of those sentences that needs to be simplified. Such long and convoluted sentences occur sometimes throughout the document, but make your manuscript difficult to read.

According to your opinion, we revised the expression of the article, using short sentences as much as possible, and adjusted the language of the full text.

Reviewer #2: Comments to the Author: 1. From the perspective of the pig industry, this article identifies the dynamic impact of environmental regulations on pig production in China, which has an academic value.

2. However, this article has some technical datils to improve.

①The variable which is utilized to measure breeding herd structure is a little simple. I do not think it can proxy the structure precisely.

The herd structure includes pigs, sows and fattening pigs. Although the article constructs the relationship among the three and makes predictions, the number of pigs in the herd structure will not affect the pig production in a certain period, but only provide breeding. For pigs, the core index is not the number of communities, but the quality of semen, which will affect the number of sows. Therefore, this paper ignores the pigs and only analyzes the proportion of sows and fattening pigs when analyzing the herd structure. In addition, sows and fattening pigs are often used as key indicators of herd structure in government documents in China, such as the application of relevant head of the National Development and Reform Commission in interviews (http://news.hexun.com/2009-06-16/118660340.html)

②The control variables in sys-GMM are the price which contains 12 years, but this article did not adjust them with price index. It may lead to estimation bias.

The empirical analysis data in this paper are reduced based on 2008, which was not explained in the previous manuscript, and 353 lines are added.

③The variable lnASF is not shown in the variables descriptive statistics table.

The variable ASF is a virtual variable of whether there has been an outbreak of African swine fever. In this modification, a statistical description of this indicator is added to the statistical description, and the previous erroneous expression lnASF is modified.

④what is the meaning of three stars on t statistics value of variable lnASF in Regression 3 in Table 6？

This is an error caused by modification. We did two processes, one was to mark "*" after coef. The other was to change the name of the variable. We didn't log the variable ASF, which is always a dummy variable. We assigned it 0 and 1 to measure the influence of African swine fever on the yield. In addition, according to your suggestion, we also explained the response in the selection of control variables, as shown in line 345.
